# Thyroid-related ophthalmopathy development in concurrence with growth hormone administration

**DOI:** 10.1186/s12902-021-00834-2

**Published:** 2021-08-19

**Authors:** Shimpei Iwata, Kenji Tsumura, Kenji Ashida, Ichiro Tokubuchi, Mutsuyuki Demiya, Miyuki Kitamura, Hiroyuki Ohshima, Mamiko Yano, Ayako Nagayama, Junichi Yasuda, Munehisa Tsuruta, Seiichi Motomura, Shigeo Yoshida, Masatoshi Nomura

**Affiliations:** 1 Division of Endocrinology and Metabolism, Department of Internal Medicine, Kurume University School of Medicine, 67 Asahi-machi, Kurume, Fukuoka 830-0011 Japan; 2 Clinical training center, Kurume University Hospital, Kurume, Fukuoka Japan; 3 Division of Endocrinology and Metabolism, Omuta City Hospital, Omuta, Fukuoka Japan; 4 Department of Pediatrics and Child Health, Kurume University School of Medicine, Kurume, Fukuoka Japan; 5 Department of Ophthalmology, Kurume University School of Medicine, Kurume, Fukuoka Japan

**Keywords:** Thyroid-related ophthalmopathy, Growth hormone, Insulin-like growth factor-I, Graves’ disease

## Abstract

**Background:**

Thyroid stimulating hormone (TSH) receptor and local infiltrate lymphocytes have been considered as major pathological factors for developing thyroid-related ophthalmopathy. Overexpression of insulin-like growth factor-I (IGF-I) receptor has emerged as a promising therapeutic target for refractory patients. However, the relationship between activation of growth hormone (GH)/IGF-I receptor signaling and development or exacerbation of thyroid ophthalmopathy has not been elucidated. Herein we describe a case that provides further clarification into the association between thyroid-related ophthalmopathy and GH/IGF-I receptor signaling.

**Case presentation:**

A 62-year-old Japanese female diagnosed with thyroid-related ophthalmopathy was admitted to Kurume University Hospital. She had received daily administration of GH subcutaneously for severe GH deficiency; however, serum IGF-I levels were greater than + 2 standard deviation based on her age and sex. She exhibited mild thyrotoxicosis and elevation in levels of TSH-stimulating antibody. Discontinuation of GH administration attenuated the clinical activity scores of her thyroid-related ophthalmopathy. Additionally, concomitant use of glucocorticoid and radiation therapies resulted in further improvement of thyroid-related ophthalmopathy. The glucocorticoid administration was reduced sequentially, followed by successful termination. Thereafter, the patient did not undergo recurrence of thyroid-related ophthalmopathy and maintained serum IGF-I levels within normal physiological levels.

**Conclusions:**

We describe here a case in which development of thyroid-related ophthalmopathy occurred upon initiation of GH administration. GH/IGF-I signaling was highlighted as a risk factor of developing thyroid-related ophthalmopathy. Additionally, aberrant TSH receptor expression was suggested to be a primary pathophysiological mechanism within the development of thyroid-related ophthalmopathy. Physicians should be aware of the risks incurred via GH administration, especially for patients of advanced age, for induction of thyroid-related ophthalmopathy.

**Supplementary Information:**

The online version contains supplementary material available at 10.1186/s12902-021-00834-2.

## Background

Thyroid-related ophthalmopathy is an autoimmune disease that reduces quality of life due to visual disturbance related to orbitopathy, and is often refractory to various therapies [1]. The thyroid stimulating hormone (TSH) receptor is considered to be a major player in the development of thyroid-related ophthalmopathy [2, 3]. Local infiltrating lymphocytes have also been implicated in the pathogenesis of this disease [3]. Glucocorticoid therapy, either orally, intravenously as steroid pulse therapy, or via orbital local injection, as well as radiation therapy are the primary therapeutic approaches employed to attenuate inflammation and prevent fibrosis in post-orbital tissue [2, 4]. However, these traditional therapies used in accordance with current guidelines sometimes fail to ameliorate ophthalmopathy, leading to persistent and/or recurrent disease.

The insulin-like growth factor-I (IGF-I) receptor in orbital organs has recently been implicated as an emerging therapeutic target for thyroid ophthalmopathy [5]. A clinical trial using an IGF-I receptor inhibitor for treatment of thyroid-related ophthalmopathy is ongoing [6]. Additionally, immune activation by the growth hormone (GH)/IGF-I has been disclosed in previous literatures [7]. In this context, involvement of GH/IGF-I signal in thyroid-related ophthalmopathy has been further elucidated. However, whether GH administration entails a risk for development or exacerbation of thyroid ophthalmopathy remains unclear.

The present case report describes a patient exhibiting thyroid-related ophthalmopathy that developed after the initiation of GH subcutaneous administration for treatment of adult GH deficiency (GHD), and improved concomitant with discontinuation of these injections [8]. The patient tested negative for TSH receptor antibody (TRAb) and mild elevation in thyroid stimulating antibody (TSAb) [9], which suggested a potential role of the GH/IGF-I system in the pathogenesis of thyroid-related ophthalmopathy (Supplemental Figure 1). Physicians should be aware of the risk for thyroid-related ophthalmopathy as a potential adverse event of GH administration.

## Case presentation

**Fig. 1:**
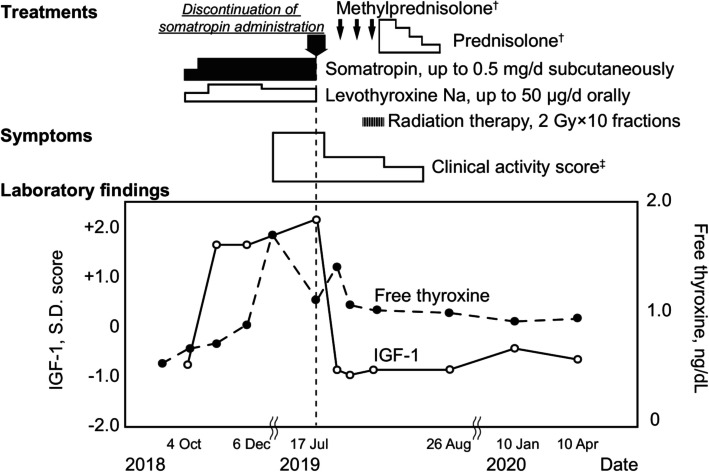
Clinical course of the present case. Thyroid ophthalmopathy was developed after growth hormone replacement therapy and was then attenuated by discontinuation of growth hormone and glucocorticoid therapy. Serum IGF-I levels were elevated > + 2 SD matched for age and sex, and decreased to within the normal range after discontinuation of somatropin administration. The open circles with solid lines and closed circles with dashed lines present SD scores of IGF-I and serum free thyroxine levels, respectively. †, methyl prednisolone was administered as three courses of corticosteroid pulse therapy and was followed by oral prednisolone. Initial dose of oral prednisolone was 20 mg/day that was gradually decreased and was then discontinued on November 22, 2019. ‡, Activity of thyroid ophthalmopathy is presented as clinical activity score. Abbreviations: IGF-I, insulin-like growth factor-I.

A 62-year-old Japanese female patient was admitted to the endocrinology center of Kurume University Hospital complaining of recent orbital pain, especially during eye movement and bilateral eyelid swellings. One year prior to her visit, she was diagnosed with severe adult GHD and secondary hypothyroidism due to low levels of both TSH and free thyroxine (T4) [10, 11], and insufficient elevation in GH and TSH levels following intravenous administration of 100 µg of GH-releasing peptide-2 (GHRP-2) or 200 mg of thyrotropin releasing hormone (TRH) (Supplemental Table 1). Thus, the patient was administered 0.5 mg/day of subcutaneous somatropin, recombinant GH, injection and 50 µg/day of oral levothyroxine. The clinical course is shown in Fig. 1.

**Fig. 2:**
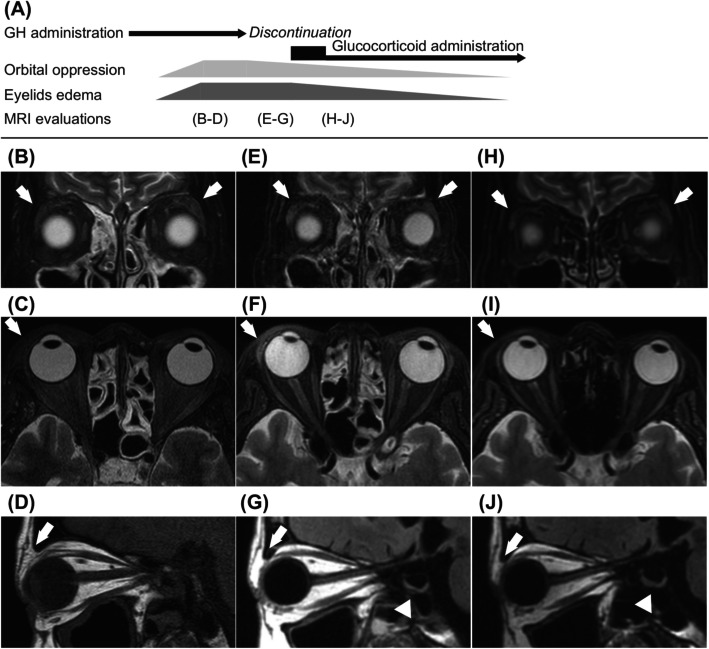
Sequential change of thyroid-related ophthalmopathy demonstrated by magnetic resonance imaging. The clinical course of growth hormone and glucocorticoid therapy, symptoms of Graves’ ophthalmopathy, and points of MRI evaluations are displayed (**A**). The bilateral lachrymal glands swelling, especially in left side (**B**), and right eyelid swelling (**C**, **D**) indicating thyroid-related ophthalmopathy after growth hormone replacement are shown. Minimal attenuation of swellings in lachrymal glands is shown after growth hormone discontinuation (**E**), while edematous change in right eyelid persisted (**F**, **G**). Glucocorticoid therapies improve further bilateral lachrymal glans and right eyelid edema (**H**, **I**, **J**). (**B**, **E**, **H**): frontal section of T2 weighted image. (**C**, **F**, **I**): horizontal section of T2 weighted image (C: short T1 inversion recovery; F and I: iterative decomposition of water and fat with echo asymmetry and least squares estimation). (**D**, **G**, **J**): sagittal section image (D: T1 weighted image; G and J: fluid-attenuated inversion recovery). Arrows indicate bilateral lachrymal gland (**B**, **E**, **H**) and right eyelids (**C**, **D**, **F**, **G**, **I**, **J**). Arrowheads (**G**, **J**) indicate pituitary gland exhibiting a mild compression form

**Fig. 3:**
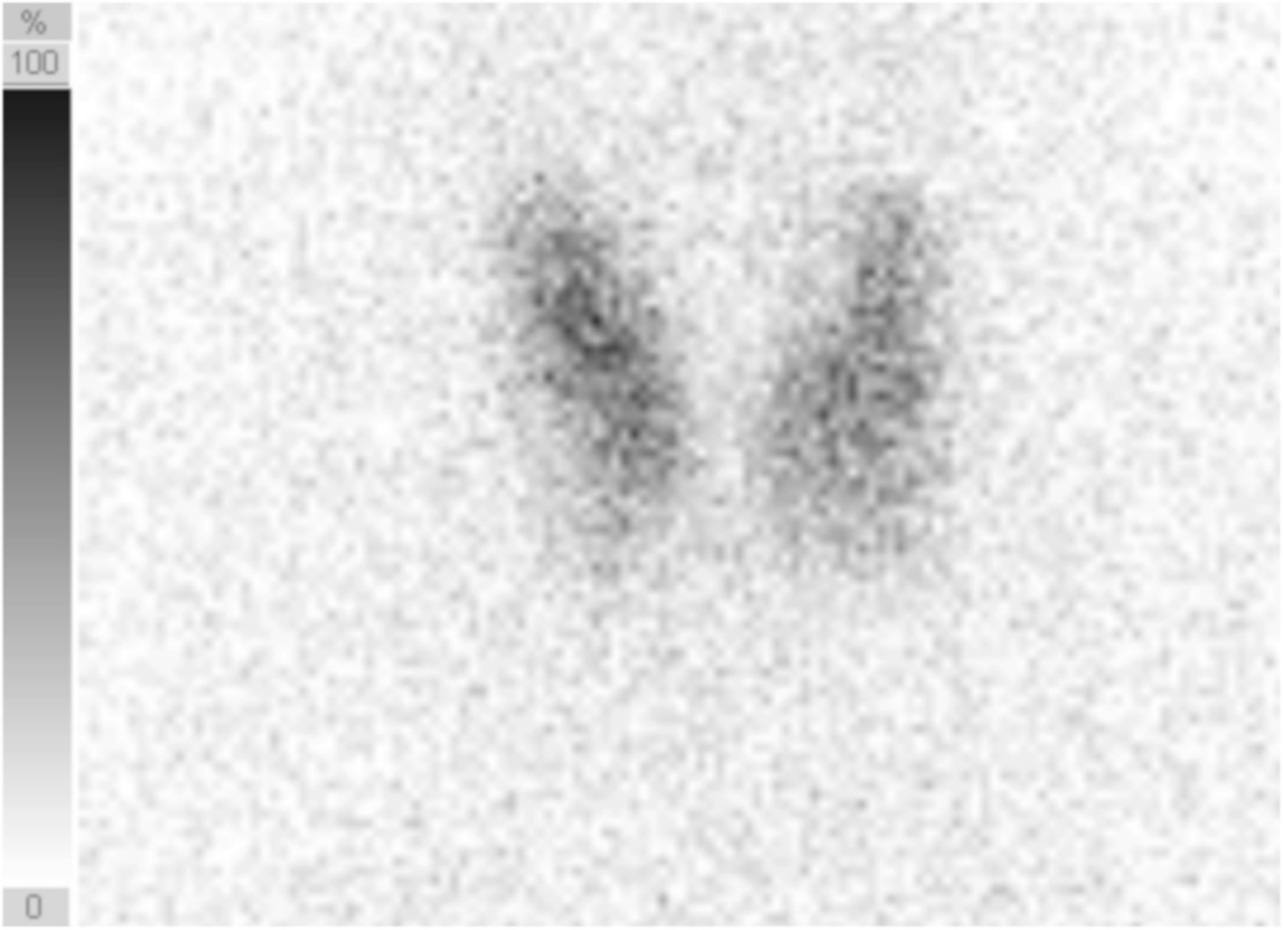
Thyroid gland uptake of ^123^I-scintigraphy. Diffuse uptake of ^123^I by 4.7 % is demonstrated under the thyrotoxicosis condition after 3 h

Upon physical examinations, bilateral swelling of the eyelids was observed. Magnetic resonance imaging (MRI) revealed swelling of bilateral lachrymal gland and right upper eyelid edema (Fig. 2), which suggested thyroid-related ophthalmopathy; however, hypertrophy of the extraocular muscles was not detected. An MRI time course was displayed sequentially at diagnosis, at 13 days after GH discontinuation, and after ophthalmopathy treatment (Fig. 2). The subject was diagnosed with thyrotoxicosis based on clinical symptoms, including sweating and palpitation, as well as laboratory findings upon admission (Table 1). We discontinued levothyroxine administration when the patient was diagnosed with Graves’ disease based on clinical findings, including positive detection of TSAb [9], increased blood flow in diffuse goiter shown by ultrasonography, and preserved diffuse ^123^I uptake by 4.7 % in the thyroid gland under thyrotoxicosis (Fig. 3). In addition, neither free thyronine nor free T4 levels decreased, and TSH remained below the detection level even following levothyroxine discontinuation. The clinical activity score (CAS) for thyroid-related ophthalmopathy was 3: positive for orbital pain, pain during eye movement, and lachrymal gland and eyelid swelling.

Somatropin, administered at 0.5 mg/day subcutaneously, was also discontinued since serum IGF-I levels were elevated to 205 ng/mL (reference range for ± 2 standard deviation [SD]: 68–196) [12]. Orbital oppression and left lachrymal gland swelling were attenuated after somatropin discontinuation (Fig. 2). Three sequential courses of corticosteroid pulse therapy (each course involved intravenous drip infusion of 0.5 g/day methyl prednisolone [PSL] for 3 days followed by 20 mg/day oral PSL for 4 sequential days) and fractional radiation therapy (20 total gray at 2 Gy/day for 10 days) were performed. CAS was improved to zero points, and orbital MRI demonstrated improvement in lachrymal glands swellings and right eyelid edema (Fig. 2). Furthermore, we were able to terminate oral prednisolone administration without recurrence of ophthalmopathy for an additional year (Fig. 1).

**Table. 1:** Laboratory findings at admission

Parameters	Value	Reference range
**Thyroid gland**		
TSH, µIU/mL	< 0.005	0.5–5.0
Free T3, pg/dL	4.2	2.3–4.0
Free T4, ng/mL	1.07	0.93–1.70
Anti-Tg Ab, IU/L	94	< 28
Anti-TPO Ab, IU/L	348	< 16
TSAb, %	152	< 120
TRAb, IU/L	1.9	< 2.0
**Growth hormone**		
Growth hormone, ng/mL	1.49	0.13–9.88
IGF-I, ng/mL (SD score^a^)	205 (+ 2.2 SD)	68–196
**Other endocrinology**		
ACTH, pg/mL	63.7	7.2–63.3
cortisol, µg/dL	10.1	6.24–18.0
LH, mIU/mL	29.7	5.72–64.31
FSH, mIU/mL	44.8	< 157.79
Estradiol, pg/mL	< 5.0	< 47.0

^a^SD score of IGF-I was calculated in accordance with LMS method [12].

Abbreviations: *ACTH* adrenocorticotropic hormone; *anti-Tg Ab* anti-thyroglobulin antibody; *anti-TPO Ab* anti-thyroid peroxidase antibody; *IGF-I* insulin-like growth factor-I; *LH* luteinizing hormone; *FSH* follicle-stimulating hormone; *SD* standard deviation; *TRAb* TSH receptor antibody; *TSAb* thyroid stimulating antibody.

## Discussion and conclusions

To the best of our knowledge, this is the first described case in which thyroid-related ophthalmopathy could be induced by GH administration. This study highlights GH/IGF-I receptor signaling as a key risk factor for thyroid-related ophthalmopathy, although aberrant TSH receptor expression is known to be a major involved pathophysiological mechanism. Physicians should be aware of the risk of GH administration for the patients with upper middle or advanced age in induction of thyroid-related ophthalmopathy.

This study demonstrated compelling evidence that GH administration can induce thyroid-related ophthalmopathy. The development and activity of ophthalmopathy are positively correlated with the levels of TSH receptor antibody [13, 14]. In this context, mild elevation of TSAb may indicate that the GH administration enhanced GH/IGF-I signaling highly contributed to thyroid-related ophthalmopathy development in this case. Subcutaneous administration of GH for one month led to occurrence of bilateral swelling of the lachrymal glands and the eyelids. Discontinuation of GH administration alone attenuated this orbital oppression, and glucocorticoid combined with radiation therapies provided further improvement (Figs. 1 and 2). Thus, excessive activity of GH/IGF-I is highlighted as a risk factor for developing thyroid ophthalmopathy. Physicians should be vigilant about the risk that thyroid-related ophthalmopathy may be induced by GH initiation, and they should adjust the GH dose to regulate IGF-I levels within normal range [12].

This study suggests that GH administration can be a key factor within thyroid-related ophthalmopathy development. The pathological cooperative signals of the TSH and IGF-I receptors in bone marrow-derived fibroblasts—which lead to the production of inflammatory molecules and hyaluronan, as well as adipogenesis in orbital organs—have been previously described [15–18] (Supplemental Figure 1). Given the IGF-I receptor antibody, teprotumumab, improved thyroid ophthalmopathy [19], it is conceivable that the activation of GH/IGF-I signaling is involved in ophthalmopathy development. The current study also suggests that Graves’ disease and aging may contribute to the overexpression of IGF-I receptor and aberrant expression of TSH receptor. Additionally, estrogens are known to antagonize the production of IGF-I in GHD patients under GH replacement therapy [20]. GH/IGF-I signaling could be modulated in post-menopausal women, as in the present case, compared to women of fertile age. Therefore, modulating factors of GH/IGF-I signaling, including menopause, should also be considered.

GH/IGF-I hyperactivity may induce or aggravate ophthalmopathy, especially in patients with a background of Graves’ disease. GH administration can exacerbate inflammation in orbital tissues via activation in autoimmunity (Supplemental Figure 1) [7]. However, thyroid-related ophthalmopathy complicated with acromegaly has not been previously reported. In children and adolescents exhibiting elevated GH/IGF-I activities, the pathophysiological mechanisms of thyroid ophthalmopathy are considered to be similar to those in adult patients [21]. However, thyroid ophthalmopathy is rare in children and adolescents [13]. Scarce evidence of thyroid-related ophthalmopathy in young people indicates that abnormal expression of the TSH receptor may be due to primary etiology, while GH/IGF-I signaling can enhance ophthalmopathy (Supplemental Figure 1). The expression levels of the IGF-I receptor within orbital tissues in patients with thyroid-related ophthalmopathy may alter with aging. Age-related decreases in local IGF-I binding protein levels may be another plausible reason for this phenomenon [22]. We could not confirm aberrant TSH receptor expression nor IGF-I receptor overexpression within the orbital tissues in our patient. To corroborate our conclusions, further case or clinical studies are warranted.

In conclusion, we describe a patient with thyroid-related ophthalmopathy that was induced by GH administration. GH/IGF-I signaling was highlighted as a risk factor of developing thyroid-related ophthalmopathy. Physicians should be aware of the risk of GH administration in patients at an advanced age who are at a risk of developing thyroid-related ophthalmopathy.

## Supplementary Information

**Additional file 1:** Supplemental **Table S1**. Pituitary gland hormone responses to pituitary stimulation tests.

**Additional file 2:** Supplemental **Figure S1**. Hypothesis of pathological mechanisms in this case.

## Data Availability

All data generated or analyzed during this study are included in this published article and its supplementary information files.

## Abbreviations

CAS: Clinical activity score
GH: Growth hormone
GHD: Growth hormone deficiency
GHRP-2: Growth hormone-releasing peptide-2
IGF-I: Insulin-like growth factor-I
MRI: Magnetic resonance imaging
PSL: Prednisolone
TRAb: Thyroid stimulating hormone receptor antibody
TRH: Thyrotropin releasing hormone
TSAb: Thyroid stimulating antibody
TSH: Thyroid stimulating hormone
T4: Thyroxine
