# Supplementary material for: Thyroid-related ophthalmopathy development in concurrence with growth hormone administration
Source: BMC Endocr Disord. 2021 Aug 19;21:168. doi: 10.1186/s12902-021-00834-2 (PMC8375170; doi:10.1186/s12902-021-00834-2)
Supplement: Supplementary file 1 — Additional file 1: Supplemental Table S1. Pituitary gland hormone responses to pituitary stimulation tests. [file 12902_2021_834_MOESM1_ESM.docx]

**Supplemental Table 1. Pituitary gland hormone responses to pituitary stimulation tests**

| **Parameters** | **Time, minutes** | | | |
| --- | --- | --- | --- | --- |
|  | 0 | 30 | 60 | 90 |
| **GHRP-2 loading test**^†1^ |  |  |  |  |
| GH, ng/dL | <0.03 | 1.24 | 0.79 | NA |
|  |  |  |  |  |
| **TRH loading test**^†2^ |  |  |  |  |
| TSH, μU/mL | 0.790 | 10.414 | 7.887 | 5.209 |
|  |  |  |  |  |
| **CRH loading test**^†3^ |  |  |  |  |
| ACTH, pg/mL | 84.6 | 184.0 | 105.0 | 58.7 |
| Cortisol, μg/dL | 16 | NA | 19.9 | 16.1 |
|  |  |  |  |  |
| **LHRH loading test**^†4^ |  |  |  |  |
| LH, mIU/mL | 17.92 | 65.76 | 67.60 | 55.61 |
| FSH, mIU/mL | 35.58 | NA | 61.58 | 58.13 |

†1, 100 μg of GHRP-2; †2, 200 μg of TRH; †3, 100 μg of CRH; †4, 100 μg of LHRH were administered intravenously.

ACTH, adrenocorticotropic hormone; CRH, corticotropin-releasing hormone; FSH, follicle stimulating hormone; GH, growth hormone; GHRP-2, growth hormone-releasing peptide-2; LH, luteinizing hormone; LHRH, luteinizing hormone-releasing hormone; NA, not applicable; TRH, thyrotropin releasing hormone; TSH, thyroid stimulating hormone.
