# Supplementary material for: Thyroid-related ophthalmopathy development in concurrence with growth hormone administration
Source: BMC Endocr Disord. 2021 Aug 19;21:168. doi: 10.1186/s12902-021-00834-2 (PMC8375170; doi:10.1186/s12902-021-00834-2)
Supplement: Supplementary file 2 — Additional file 2: Supplemental Figure S1. Hypothesis of pathological mechanisms in this case. [file 12902_2021_834_MOESM2_ESM.docx]

**Supplemental Figure 1. Hypothesis of pathological mechanisms in this case**

Infiltration of bone marrow derived fibroblast to the orbital tissues, and ectopic expression of TSH receptor and overexpression of IGF-1 receptor on this fibroblast were suggested for the primary origin of Graves’ ophthalmopathy. GH administration, which activates IGF-1 production, may lead to the enhancement of aberrant TSH receptor signaling.
